# The Whole-of-Person Retention Improvement Framework: A Guide for Addressing Health Workforce Challenges in the Rural Context

**DOI:** 10.3390/ijerph17082698

**Published:** 2020-04-14

**Authors:** Catherine Cosgrave

**Affiliations:** Department of Rural Health, Faculty of Medicine, Dentistry and Health Sciences, University of Melbourne, Docker St, Wangaratta, VIC 3677, Australia; ccosgrave@unimelb.edu.au; Tel.: +61-405-110-897

**Keywords:** rural health workforce, framework, retention, turnover, Australia, allied health, nursing, medical professionals

## Abstract

People living in rural places face unique challenges due to their geographic isolation and often experience poorer health outcomes compared to people living in major cities. The struggle to attract and retain an adequately-sized and skilled health workforce is a major contributing factor to these health inequities. Health professionals’ decisions to stay or leave a rural position are multifaceted involving personal, organisational, social and spatial aspects. While current rural health workforce frameworks/models recognise the multidimensional and interrelated influences on retention, they are often highly complex and do not easily support the development of strategic actions. An accessible evidence-informed framework that addresses the complexity but presents the evidence in a manner that is straightforward and supports the development of targeted evidence- and place-informed retention strategies is required. The ‘Whole-of-Person Retention Improvement Framework’ (WoP-RIF) has three domains: Workplace/Organisational, Role/Career and Community/Place. The necessary pre-conditions for improving retention through strengthening job and personal satisfaction levels are set out under each domain. The WoP-RIF offers a person-centred, holistic structure that encourages whole-of-community responses that address individual and workforce level needs. It is a significant response to, and resource for, addressing avoidable rural health workforce turnover that rural health services and communities can harness in-place.

## 1. Introduction

Compared to people who live in major cities, people living in rural and remote places face unique challenges and often experience poorer health outcomes due to their geographic isolation [1,2]. A major contributing factor to these health inequities is the struggle to attract and maintain an adequately-sized and skilled workforce to meet rural communities’ health care needs [2]. Under-resourcing contributes to rural health workforce shortages and is a self-perpetuating problem. High staff turnover and long-term unfilled positions limit the health services that can be provided. This simultaneously results in heavy workloads for existing staff, which negatively impacts upon their job satisfaction, leading to further staff turnover [3,4]. Given their large land masses and small, broadly-dispersed populations with high proportions of Indigenous peoples, high income countries like Australia and Canada face additional challenges meeting their rural populations’ health needs. In Australia, approximately seven million people (28% of the population) live in rural and remote areas, and health workforce shortages have been strongly attributed to unaddressed healthcare needs [1,3,5,6]. In an attempt to address these rural workforce challenges, the Australian government has been implementing a range of policy measures for more than twenty years. In particular, the government has invested in training and development to increase the supply of health professionals, and in university-based research to better understand rural health workforce challenges and, thus, strengthen the evidence base for addressing these issues [7].

In the literature, job satisfaction is strongly correlated with increased retention [8]. Retention has also been found to be contingent on the extrinsic rewards provided by the employer (e.g. salary and work conditions) and the intrinsic rewards that come from within the individual, which are derived from the role and the work being performed (e.g., degree of autonomy and/or challenge) [9]. For rural and remote allied health professionals, the most cited extrinsic factors with a negative influence on retention are lack of professional development opportunities, professional isolation and insufficient supervision, while the most cited intrinsic factors with a positive influence on retention are autonomy and community connectedness [9]. However, recent analyses posit that health professionals’ decisions to stay or leave a rural health position (retention/turnover) are complex and influenced by ‘a myriad of highly interactive dimensions within personal, organisational, social and spatial domains’ [10].

Factors that influence retention for the rural health workforce can be broadly categorized into three domains: organisational (or workplace); role (including profession and career development opportunities); and personal (including individual characteristics, spousal and family support, social aspects and lifestyle interests) [11,12,13,14]. In the main, retention studies have focused on the influence of organisational and role conditions, with far less research being undertaken to strengthen understanding of the inter- and intra-personal determinants. This is despite the fact that psychosocial and personal factors are increasingly being found to significantly influence retention [13,15,16,17]. Notwithstanding this uneven focus in current research, the identification of factors that influence rural health workforce retention have been important for building understanding regarding the complex interplay between the domains and the need for development of effective strategies in all three domains [18]. More recent research into these three domains has also assisted in the identification and understanding of factors that can be influenced (e.g., training and education, management support, work conditions) and those that are difficult or cannot be influenced (e.g., lifestyle preferences) through policy or community actions [18]. Within rural health workforce research, this more nuanced understanding of impacting factors has led to the widespread adoption of human resource management terminology and the use of the turnover taxonomy classifications ‘voluntary’, ‘involuntary’, ‘avoidable’ and ‘unavoidable’ [12,19,20,21].

The retention research has also underpinned the development of some frameworks and models for understanding rural health workforce retention [18,21,22,23,24]. While most of these existing frameworks and models recognise the multidimensional and interrelated influences on retention, they have tended to be highly complex, including comprehensive lists of the range of factors involved [18,22,23,24]. While such models likely assist in building understanding around the complexity of the rural health workforce issue, they do not necessarily support the development of place-based strategic actions.

**Figure 1:**
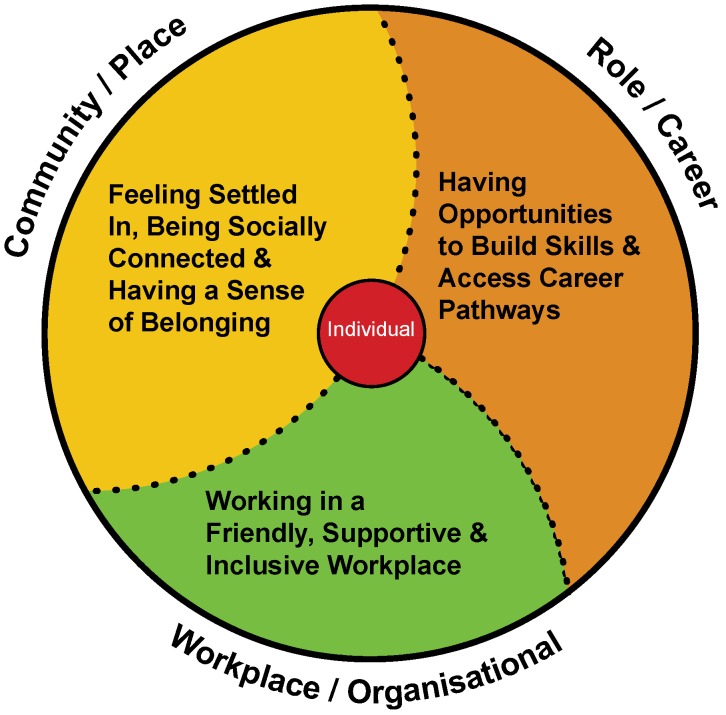
The ‘Whole-of-Person’ Retention Improvement Framework.

There remains a critical need for an evidence-based framework to better address the needs of individual health workers and the collective rural allied health, nursing and medical workforces. This article provides a new, accessible, solution-focused, holistic, person-centred and evidence-based conceptual framework—the ‘Whole-of-Person Retention Improvement Framework’ (WoP-RIF)—that can be used in a variety of different contexts to direct action/s at the local workplace, organisational and community levels for improving workforce retention [see Figure 1]. This framework has been formulated specifically to support Australia’s rural health service executives and line managers, rural communities, and governing bodies to develop effective strategic actions to improve rural health workforce retention. As such, it prioritises Australian research over international, although both are referenced. The ‘rural’ focus includes regional, rural and remote places, meaning anywhere outside major cities. In this article, the use of the term ‘rural’ should be considered to cover all types of rural settings unless otherwise specified. In the rural health workforce literature studies, retention (the average duration of stay of health professionals) and/or turnover (extent of workplace or workforce flux) terminology/measures are commonly used and both are employed interchangeably in this article.

## 2. Materials and Methods

### Development of the Framework

The WoP-RIF [see Figure 1] draws on my doctoral research, which sought to identify the full range of ‘life’ factors affecting turnover intention of rural-based allied health and nursing professionals [25]. ‘Turnover intention’ (an individual’s thoughts about leaving and intention to quit) is often used as a broad indicator of staff satisfaction and to estimate possible future workforce turnover [26]. I employed a constructivist grounded theory methodology for my doctoral research, and developed a substantive theory explaining turnover intention based on in-depth interviews undertaken with 26 allied health and nursing professionals working in public sector community mental health services operating in rural and remote New South Wales (NSW), Australia [17]. The interviews explored workplace conditions, professional development and career advancement opportunities and social and personal conditions.

The resulting turnover intention theory was holistic and person-centred. It proposed that individual health professionals’ decisions to stay or leave their rural position were determined by the extent of the gap between their personal and professional expectations and their current job and rural living experience [17]. The extent of an individual’s professional (defined in the study as including both job and career factors) and personal expectations were assessed by satisfaction levels [17]. The factors found to most strongly influence professional satisfaction were: the scope of the role; the quality of workplace relationships; the extent of access to continuing professional development; and career advancement opportunities. Professional satisfaction levels were found to be strongly affected by the time in the job [17]. While all participants experienced a difficult initial adjustment upon starting work in a rural place, the duration and severity was found to be affected by the extent of professional experience, with ‘early career-beginners’ (those with three years or less work experience) experiencing the hardest and longest transition—between 12 months and two years [17]. The influencing factors on personal satisfaction were an individual’s sense of belonging towards the town they lived and worked in and their assessment of the town’s ability to meet their (and any other family member’s) future life aspirations [17]. Not surprisingly, significant differences were found in levels of personal satisfaction between ‘locals’ and ‘non-local newcomers’. While locals commonly expressed a strong sense of belonging, newcomers described feelings of alienation and social disconnection, especially in the first 12 months of moving to the town, but reported that their sense of belonging-in-place usually increased over time [17]. 

The turnover intention study identified that life stage rather than rural origin was the strongest influence on the decision to stay and thus workers in ‘middle adulthood’ were the most likely to stay, while those in ‘early adulthood’ were the least likely [17]. The decision to stay or leave was found to be made fairly early on in employment, most commonly by 12–18 months, but in some cases earlier than this [17]. The study’s findings were drawn on to develop a turnover intention risk matrix with three categories of vulnerability (high, moderate, minimal) [17], the most vulnerable category of rural health professional workers being ‘early career beginners’ and ‘non-local newcomers’ and the least vulnerable ‘experienced’ health professionals in ‘middle adulthood’ [17]. The factors that were identified as impacting turnover intention in the study were found to be well supported in the existing rural health retention research [17,25]. The employment and rural-living concepts underpinning the turnover intention theory, such as transition to practice, adaptation to rural living, health teams and organisational dynamics, social dynamics of rural communities, and influences of life stage, were also well supported in the extant literature [17,25].

The WoP-RIF was based on the findings from this doctoral research study and were cross-referenced and considered in relation to an extensive body of rural health retention literature, both peer-reviewed and grey literature, that I collected, synthesised, analysed and evaluated over eight years. From hereafter, my doctoral study will be described as the/my ‘NSW turnover intention study’. A full description of the WoP-RIF is provided in the next section.

## 3. Results

### 3.1. The Framework

The WoP-RIF has three domains: Workplace/Organisational, Role/Career and Community/Place [see Figure 1].

The necessary preconditions for improving retention through strengthening job and personal satisfaction levels are set out under each domain. These preconditions are: working in a friendly supportive, inclusive workplace (Workplace/Organisational); having opportunities to build skills and access career pathways (Role/Career); and feeling settled in, being socially connected, and having a sense of belonging (Community/Place). The individual is placed at the centre of the WoP-RIF, signifying that each person’s experience and needs are highly individual and when developing recruitment and retention strategies, line managers, organisations and/or communities need to understand and address this diversity. The dotted lines between signify the interrelatedness and porous nature of the division between the three domains. For example, socialisation in the Community/Place domain is also an important process operating in the Workplace/Organisation and Role/Career domains. An in-depth discussion of the major influences on job and personal satisfaction identified in the rural health workforce literature and underpinning the identified preconditions in each WoP-RIF domain now follows. A summary of the key influences and elements on job and personal satisfaction of rural health professionals is provided in Table 1.

### 3.2. Workplace/Organisational Domain

#### 3.2.1. Workplace

Rural health workforce stability and sustainability have been identified in rural health workforce research as being strongly impacted by the quality of workplace relationships, especially the relationship workers have with their line manager/supervisor [4,14,17]. In human resources management studies (research on people and workforce management), the quality of the employee–manager relationship is termed the ‘leader–member exchange’ relationship, which has been found to influence employees’ organisational commitment [27]. Onnis, in her northern Australia health workforce retention study, identified that for employees, the quality of the employee–manager relationship was determined by the extent to which an employee felt supported by their line manager [4]. ‘Feeling supported’ involved line managers effectively communicating and demonstrating real understanding of the challenges of working in a remote setting [4]. Poor manager communication in the rural health workforce literature has been widely linked to job dissatisfaction [12,28,29]. My NSW turnover intention study strongly supports Onnis’ findings, identifying job satisfaction as strongly linked to the extent rural health workers felt valued by their line-manager. This was explained as a manager demonstrating understanding of ‘the challenges and demands of their job and appreciating their efforts’ [17] (p. 9). Team dynamics, which is the level of team cohesion and the extent to which an employee feels supported by their colleagues, was also identified in the rural health workforce research as an important influence [12,14,29]. Participants in my NSW turnover intention study discussed the team dynamic being strongly influenced by the mix of experience levels and professions, age ranges and gender composition in the team and that staffing changes, especially in small teams, could change the dynamic significantly, sometimes for the better, often times for the worse [17]. 

Participants in my NSW turnover intention study also discussed that feeling supported by their line manager included their manager making assessments regarding ‘fit’ when recruiting new staff. This was explained as managers considering resourcing constraints and current team composition [25]. In terms of workforce sustainability, Onnis also highlighted the importance of having recruitment processes that allow for ‘person-fit’ considerations when selecting staff [4]. In the human resources management field ‘person-environment-fit’ selection is defined as assessing applicants’ fit with the job, with the organisation and with the work group and giving consideration to their qualifications, work experience, values and interpersonal attributes [30]. Another important management practice relating to employee job satisfaction was employees being given a comprehensive orientation [12]. Some participants in my turnover intention study discussed the long-lasting negative impact of having received no or very little orientation and describing, due to staff resourcing constraints, that they were expected to just jump in and get on with the job from day one [25].

#### 3.2.2. Organisational

In a systematic review of effective retention incentives for rural and remote health workers, an important retention factor is staff perceiving the ‘organisation as being managed efficiently and strategically’ [12,31]. Both Onnis’ remote northern Australia study [4] and my rural and remote NSW study [17] found retention was associated with the extent to which staff felt they worked in a ‘supportive’ organisation: ‘when you’ve got an executive and health service that supports you…it makes you want to stay’ [4] (p. 16). Onnis’ remote workforce study found that line managers were often perceived as representing the ‘organisation’ and, thus, their level of support was equated with what the organisation provided [4]. In my NSW turnover intention study, organisational culture and staff interface with the health service’s executive and senior management impacted participants’ job satisfaction [25]. The participants in my study all worked for NSW Health (the state government organisation responsible for public health care of NSW residents) and organisational policies and procedures sent from regional or central offices were often described as demonstrating little understanding of the day-to-day realities of working in rural and remote-based services [25].

Frustration among health professionals working in an organisational culture where there was constant change and restructuring has been associated with turnover [32]. In my NSW turnover intention study, many participants discussed feeling frustrated and exhausted from working in an environment of constant change and, in particular, the negative impacts of restructures, which were discussed as rarely resulting in improvements to the service [25]. Participants spoke of organisational and procedural changes being implemented without consultation or, if they were consulted, that it was tokenistic and their feedback was usually ignored. This contributed to the existence of a pervasive ‘them-and-us’ culture and staff commonly resisting organisational (meso level) and/or procedural (micro level) changes [25].

In Onnis’ northern Australia study, the way in which work was conducted (work systems) was identified as contributing to staff’s view of management practices (micro) and organisational culture (meso) [4]. In Onnis’ study, access to suitable affordable accommodation, safe work conditions and regular leave were identified as being important work conditions for remote contexts [4]. Various studies have argued that ‘work systems’ need to suit the particular work environment and that local managers need to be able to develop employment policies that are responsive to the local context [3,4].

### 3.3. Role/Career Domain

#### 3.3.1. Role

Both my research and other health workforce literature recognise that health workers are firmly committed to their professional identity and their job satisfaction is strongly impacted by the extent to which they have opportunities to engage with other discipline-specific health professionals [33,34,35]. Having opportunities to engage with your ‘own-tribe’ is particularly important in early career when professional identity is still forming [36]. Profession-specific engagement can occur either formally (through continuing professional development (CPD) activities and clinical supervision) or informally (through mentoring and networking activities) [9]. It is also well recognised that, in rural and remote health settings, there are significant barriers (budgetary, time, staffing constraints) for creating profession-specific engagement opportunities. Rural health workforce studies, including mine, have found that this is usually a more substantial issue in small rural or remote services compared to regional services and presents the greatest challenges for allied health professionals, given their more limited staff numbers [34,37]. Onnis’ northern Australia study found that, in contrast to health workers’ strong commitment to their profession, commitment to their employer was generally weak and, thus, in places where employer choice existed, dissatisfied employees tended to just change their employer but often stayed working in the same professional role and living in the same community [4]. Both Onnis and I recommended that—to improve rural health workforce retention—employers need to develop human resources policies that support health professional staff to continue to develop their profession-specific identity [4,25]. I also recommended that NSW Health provide additional budgetary resources to assist rural staff to overcome barriers for undertaking profession-specific CPD activities, given most courses are metro- or regionally-delivered (e.g. costs associated with travel, accommodation and backfilling staff) [25]. In addition to face-to-face training, well-designed technology-enhanced training can be an effective measure for addressing rural health workers’ need for regular professional engagement [38].

#### 3.3.2. Career

In rural health workforce literature, a commonly cited reason for health professionals leaving their rural positions is lack of career opportunities [12,13,39]. In my NSW turnover intention study, having opportunities to advance and pursue professional interests was associated with job satisfaction. Participants described various types of career opportunities including: occasions for secondment to other positions at the same grading level and ‘acting’ in senior positions; providing clinical supervision to lower grade staff; and chances for promotion or regrading [25]. In Chisholm et al.’s study measuring rural allied health workforce turnover and retention in health services in Western Victoria, Australia, career grade was found to be a significant factor for retention [5]. The authors argued that, given the small size of allied health workforces in rural and remote health services, there are likely to be particularly limited opportunities for career advancement [5]. Given both the limited opportunities for career advancement and high turnover of nursing and allied health professionals in small rural and remote settings, investing in grow-your-own workforce policies is recommended for building a sustainable workforce [40,41]. Further, there is now strong evidence that offering health professional training in rural and remote environments leads to increased retention as well as ensuring fit-for-practice health professionals [40].

### 3.4. Community/Place Domain

#### 3.4.1. Community

In a scoping review investigating the role of community engagement in improving recruitment and retention of healthcare workers in rural and remote areas, ‘community engagement’ was defined as people in a community involved in issues that affect them [42]. A study investigating the participation of two rural communities in Queensland, Australia, in the development of community action plans to improve recruitment and retention of general practitioners(GPs) found that, despite the communities having different physical and social characteristics, the strategic conclusions made and the action plans created were similar [43]. Some of the common recruitment and retention strategies formulated by the two communities were: forming a liaison committee involving all stakeholders; developing information packages for prospective GPs; developing a welcome process that helps GPs and their families settle in; addressing the quality and appropriateness of housing; and considering spouses’ education and employment needs. Two primary reasons for involving rural communities in the planning process for recruitment and retention of health workers have been identified by Urquhart [42]. These are: (1) communities possess the local and historical knowledge for planning a well-tailored intervention; and (2) community involvement fosters a sense of responsibility and commitment to the successful recruitment of health workers and their integration into the community [42].

In a European Union funded rural health recruitment study entitled “Recruit & Retain—Making it Work” (2014–2018) involving five far northern countries—Canada, Sweden, Scotland, Norway and Iceland—the collaborators contend that the ultimate goal of community engagement is sustainable community ownership of the strategies developed and implemented [38]. Improved literacy about the health workforce among all stakeholders is arguably needed to support improved health workforce recruitment and retention and for the development of ‘whole-of-community’ strategies [44]. This includes stakeholders directly involved in the delivery, receipt and organisation of healthcare (e.g., local GPs, health managers and staff, community health facilitators, patients and carers, workforce agencies), as well as those in the broader community (e.g., local government, elected councillors, local businesses, schools, cultural and recreation groups) [44]. With improved health workforce literacy, new workers’ need for ‘whole-of-person’ support could be taken up by rural communities; thus, reducing the growing and unsustainable burden on workforce agencies [44].

#### 3.4.2. Place

In a rural health workforce paper investigating the conceptualisations of place and belonging in workforce retention, the authors argue that an authentic sense of place is experienced when individuals have a sense of belonging-in-place and that ‘place’ is an intrinsic part of any individual’s identity [10]. Other studies have identified that when sense-of-belonging is missing, feelings of loneliness, social isolation and alienation ensue [45]. This experience has been described as being/feeling ‘out of place’ [46]. The universality of feeling alienated when relocating to an unfamiliar place for work was confirmed in my NSW turnover intention study [17]. The ‘newcomer’ health worker participants commonly discussed experiencing alienation and social disconnection in the first 12 months of moving to a rural town where they now lived for work, indicating that during this period they felt ‘out of place’ [17]. Establishing meaningful connections in-place, both at work and in community, was termed by Onnis as ‘community embeddedness’ [4]. In other health workforce relocation-focused studies, including my own, community embeddedness has been described as a social process that often takes place over several years [17,47,48,49,50]. People who relocate to a rural place for work and have persistent feelings of social alienation and loneliness will always leave, irrespective of whether the position is financially beneficial [51]. 

As mentioned above, my NSW turnover intention study found that social disconnection and loneliness were experienced more intensely by those ‘newcomers’ who were in early adulthood. Early adulthood is generally accepted to be early–mid 20s and is a stage of life focused on career building, finding a life partner, social activities and seeking new experiences [52,53,54]. My NSW turnover intention study identified that being un-partnered also increased the experience of loneliness, while having a partner or children appeared to be protective [17]. This has been identified in other studies and explained as a result of the broader social net that family provides for making friends [17,55].

Rural health workforce retention studies that focus on ‘newcomer’ workers argue that it is essential to develop social connection through a family lens [47,56]. In a study investigating the recruitment and retention of physicians in rural Alberta, Canada, which was undertaken from both the physicians’ and spouses’ perspectives, the authors found that the decision to relocate to a rural community was mutual and couples commonly assessed a prospective town for whether it was a good place to raise a family [57]. The key factors identified as influencing retention were whether the town was able to provide the spouse with employment and the social impact of the rural move on the spouse (i.e., whether they had been able to successfully integrate into the community) [57]. 

My NSW turnover intention study found that newcomers who decided to ‘stay’ living in the town and working in their current job were mostly in middle adulthood and were involved in raising families or engaged in planning one [17]. Middle adulthood generally occurs from the late 20s onwards and is characterized as a more settled, less adventurous time of life (compared to early adulthood) and strongly focused on family and work integration [52,53,54]. The ‘newcomer-stayers’ in my NSW turnover intention study all discussed experiencing a growing attachment to place and sense of belonging, which was facilitated by the development of social relationships in-place [17]. My study found that the ‘newcomer-stayers’ made a conscious decision (with their partners, if relevant) about the rural place they were living and the local community in regard to its ability to meet their and any significant other’s future life aspirations [17]. Similarly, a USA study on the influence of practice location choices of rural-based primary care physicians over their life course found that living a happy and satisfying life was an important factor for retention [47]. Other rural retention studies have also identified life stage as being an important influence on the decision to stay [9,13,58,59,60,61].

The challenge for newcomers in forming meaningful social connections, and its primary importance for medium-long-term retention, has been identified in other rural health workforce studies [13,48,59], as well as studies investigating the relocation experience of workers (and their families) in other sectors [62,63]. In a qualitative study investigating spouses’ experiences of frequent relocations due to their partner’s employment in the mining industry, all the participants described their biggest challenge as being ‘making friends and social activities’ [62] (p. 278). In the same study, a key aspect of a ‘good place’ to live was identified as ‘whether it was welcoming or accepting of newcomers’ [62] (p.281). Interestingly, in this mining study, the towns with transient populations were described as being easier to live in and meet people. This supports findings in other studies, including my own, that newcomers generally make friends with other newcomers, not local people [28,55,56]. In my NSW turnover intention study, newcomers described their exchanges with local residents as being friendly but rarely extending beyond surface level exchanges [56]. Newcomer participants’ explanation for this was that locals already had well-formed social connections and thus did not feel any real need to step outside of these. Some newcomer participants discussed thinking that if rural communities wanted to retain newcomer professionals, community members need to take a more active role in supporting newcomers to make friends:

X *[Town’s name] is known as a closed community and people say it’s really hard to get a leg in there socially. Then perhaps you reap what you sow, if you are a closed community you’re going to have trouble keeping people around*.

[25]

In the same study, one participant proposed that local councils are well-placed to take a lead role in running social activities for newcomers:

*In X [Town’s name] we had an open night charity thing, open to everybody but particularly for doctors and teachers and ambos, police. And every month, we’d have a trivia night, a way to meet like-minded professionals. They were brilliant nights, lots of fun and a way to get to know people outside the work context*.

[25]

The importance of including spouses in any community efforts to facilitate integration and connection of newcomer workers has also been recognised in retention focused studies [47].

## 4. Discussion

### 4.1. A New Framework 

The extant rural health workforce research has, to date, primarily focused on sustainable healthcare service delivery models and, thus, the research has heavily focused on improving workforce supply of different sized rural communities for the ‘optimal’ organisation of the workforce in these different environments [64]. By drawing on a number of approaches and from different bodies of literature and fields of study, including human resource management, rural sociology, psychology, environmental psychology, human geography and community development, the WoP-RIF offers a new but complementary approach for considering rural workforce retention challenges and reducing voluntary turnover. In particular, the WoP-RIF utilises human resource management, place and belonging and community engagement approaches and concepts, all of which are newly-emerging for addressing rural health workforce challenges. Until Onnis’ recent northern Australia rural health workforce research study utilising a human resources management methodology, there was a dearth of peer-reviewed human resources management literature. This is despite the potential benefits of a human resources approach for addressing workforce challenges being recognised a decade prior [65]. 

Onnis draws on the Harvard Analytical Framework for human resources approach to support her analysis [4]. This framework takes a holistic approach to human resources management and includes five components: stakeholder interests, situational factors, human resources policy choices, human resources outcomes—immediate (e.g., retention, cost effectiveness) and human resources outcomes—long-term (individual wellbeing, organisational effectiveness and societal wellbeing) [66]. Similarly, while ‘placed-based social processes’ have increasingly been recognised as influencing rural health workforce retention, especially for newcomer workers, until fairly recently there was little research undertaken investigating how these operate and influence retention [56]. Research undertaken by Malatzky, Gillespie and myself [10,56] examining the influence of place-based social processes on turnover, argues that operational concepts such as sense of place, place attachment and belonging-in-place offer potential solutions for rural workforce challenges, especially when applied in person-centred approaches [10]. In the Recruit & Retain—Making it Work study, the need for community engagement was identified as an ‘essential’ strategic element for the recruitment and retention of the ‘right’ professionals to achieve a ‘sustainable’, ‘fit-for-purpose’ workforce [38]. The emphasis on community engagement was premised on the basis that every place is unique, and these place-specific realities must be understood and integrated into the planning and implementation of all workforce strategies to be successful [38]. This approach is captured in the study under its often-used slogan, ‘Not about us, without us’, meaning by rural communities, in rural communities, for rural communities [38].

### 4.2. The WoP-RIF in Review

The WoP-RIF offers a person-centred, holistic structure through which to understand the complex interplay of personal, organisational, social and spatial aspects recognised in the literature as influencing health professionals’ decisions to stay or leave a rural position. While earlier rural health retention frameworks/models have assisted in strengthening understanding of the complexity of the rural health workforce problem, because of their complexity they have not supported the straightforward development of evidence-based, strategic retention strategies. Another distinguishing aspect of the WoP-RIF, compared to earlier retention frameworks/models, is the equal weighting given to the Community and Place domain as Workplace/Organisational and Role/Career influences. While the Community and Place domain has relevance for all staff, the need for retention strategies in this domain is primarily for non-local newcomers. ‘Locals’ commonly experience a strong sense of belonging-in-place; thus, making a case for substantial investment in grow-your-own workforce strategies for addressing rural workforce challenges. The WoP-RIF encourages consideration of the unique organisational and community context that health services are being delivered in. Such an approach supports place-based planning, community engagement and innovation through co-designed bottom-up planning. These are all approaches increasingly being called on to develop effective recruitment and retention strategies in the rural context [67]. Analyses of the WoP-RIF’s utility for improving retention were undertaken through an allied health workforce retention improvement project with two Victorian rural public health services in 2018–2019, and the outcomes and conclusions drawn from this research are forthcoming. Further conceptual work is also being undertaken with co-researchers Malatzky and Gillespie to address the need for environmental experiences and events that are shaped by rurality in specific ways to be accounted for and accommodated within the WoP-RIF. 

## 5. Conclusions

After more than 20 years of intensive research investigating the rural health workforce retention problem, the evidence is in. The problem is now well understood, and the impacting factors clearly identified. However, despite this strong evidence base, rural health services and their communities remain unclear about what to do—the actions they can or should take to improve retention of their health professionals. In part, this is because the retention frameworks and models that have been available are overly complex and thus have not supported meaningful action. What is urgently needed is an evidence-informed framework that is accessible, addresses the complexity but presents the evidence in a manner that is straightforward to support the development of whole-of-community, targeted, evidence- and place-informed retention strategies. The WoP-RIF clearly articulates the pre-conditions for workforce retention and offers a new approach for addressing health workforce retention in rural contexts. The WoP-RIF encourages evidence-informed, whole-of-community responses that address both individual and workforce level needs and, as such, is a significant response to, and resource for, addressing avoidable rural health workforce turnover that rural health services and communities can harness in-place. 

## Figures and Tables

**Table 1:** Influences and key elements on job/personal satisfaction drawn from the rural health workforce literature—categorised under the relevant Whole-of-Person Retention Improvement Framework (WoP-RIF) domain.

WoP-RIF Domains	Major Influences on Job/Personal Satisfaction	Key Elements
Workplace	High quality workplace relationships with line manager and in team	When recruiting, line managers give consideration to person-environment-fit and team dynamics New staff receive a comprehensive orientation
Organisational	Organisation managed efficiently and strategically	Staff feel supported and understood, and are consulted by executive and senior management Extent of change (restructuring and new policies and procedures issued from head office) Systems and work conditions are appropriate for the work environment
Role	Opportunities to engage with other discipline-specific health professionals and governing bodies	Staff have regular access to continuing professional development, clinical supervision, mentoring and networking Resourcing barriers are addressed
Career	Opportunities for career development/advancement	Staff have opportunities for secondment to other roles at same grade level and for ‘acting’ in senior positions Staff have opportunities to provide clinical supervision to lower grade staff Staff have the possibility of promotion and regrading
Community	Community involved in the planning and implementation of recruitment and retention strategies	Community is involved in the development and implementation of recruitment and retention strategies to ensure they are well-tailored for the context Community is involved and invested in the successful recruitment and integration of newcomer staff
Place	Experience a sense of belonging-in-place	Staff have opportunities to engage in activities well-suited to their life stage and personal interests Strategies are in place to assist new staff and their family members to feel welcome and support adjustment Town residents are welcoming and accepting of newcomers Social opportunities are offered by community organisations and groups to support the integration of newcomers

## References

[1] Australian Institute of Health and Welfare Rural & Remote Health—Web Report. https://www.aihw.gov.au/reports/rural-health/rural-remote-health/contents/rural-health.

[2] World Health Organization (2010). Increasing Access to Health Workers in Remote and Rural Areas Through Improved Retention: Global Policy Recommendations.

[3] Wakerman J., Humphreys J., Russell D., Guthridge S., Bourke L., Dunbar T., Zhao Y., Ramjan M., Murakami-Gold L., Jones M.P. (2019). Remote health workforce turnover and retention: What are the policy and practice priorities?. Hum. Res. Health.

[4] Onnis L.A. (2019). Human resource management policy choices, management practices and health workforce sustainability: Remote Australian perspectives. Asia Pac. J. Hum. Res..

[5] Chisholm M., Russell D., Humphreys J. (2011). Measuring rural allied health workforce turnover and retention: What are the patterns, determinants and costs?. Aust. J. Rural Health.

[6] National Rural Health Commissioner (2019). Discussion Paper for Conultation: Rural Allied Health Quality, Acess and Distribution. Options for Commonwealth Government Policy Reform and Investment.

[7] Mason J. (2013). Review of Australian Government Health Workforce Programs.

[8] Griffeth R.W., Hom P.W., Gaertner S. (2000). A meta-analysis of antecedents and correlates of employee turnover: Update, moderator tests, and research implications for the next millennium. J. Manag..

[9] Campbell N., McAllister L., Eley D. (2012). The influence of motivation in recruitment and retention of rural and remote allied health professionals: A literature review. Rural Remote Health.

[10] Malatzky C., Cosgrave C., Gillespie J. (2019). The utility of conceptualisations of place and belonging in workforce retention: A proposal for future rural health research. Health Place.

[11] Brown P., Fraser K., Wong C., Muise M., Cummings G. (2013). Factors influencing intentions to stay and retention of nurse managers: A systematic review. J. Nurs. Manag..

[12] Buykx P., Humphreys J., Wakerman J., Pashen D. (2010). Systematic review of effective retention incentives for health workers in rural and remote areas: Towards evidence-based policy. Aust. J. Rural Health.

[13] Keane S., Lincoln M., Smith T. (2012). Retention of allied health professionals in rural New South Wales: A thematic analysis of focus group discussions. BMC Health Ser. Res..

[14] Scanlan J.N., Still M., Stewart K., Croaker J. (2010). Recruitment and retention issues for occupational therapists in mental health: Balancing the pull and the push. Aust. Occup. Ther. J..

[15] Campbell N., Eley D.S., McAllister L. (2016). How do allied health professionals construe the role of the remote workforce? New insight into their recruitment and retention. PLoS ONE.

[16] Gillespie J., Redivo R. (2012). Personal-professional boundary issues in the satisfaction of rural clinicians recruited from within the community: Findings from an exploratory study. Aust. J. Rural Health.

[17] Cosgrave C., Maple M., Hussain R. (2018). An explanation of turnover intention of early-career nursing and allied health professionals working in rural and remote Australia: Findings from a grounded theory study. Rural Remote Health.

[18] Schoo A.M., Stagnitti K.E., Mercer C., Dunbar J. (2005). A conceptual model for recruitment and retention: Allied health workforce enhancement in Western Victoria, Australia. Rural Remote Health.

[19] Abelson M.A. (1987). Examination of avoidable and unavoidable turnover. J. Appl. Psychol..

[20] Russell D., Wakerman J., Humphreys J. (2013). What is a reasonable length of employment for health workers in Australian rural and remote primary healthcare services?. Aust. Health Rev..

[21] Onnis L.A., Pryce J. (2016). Health professionals working in remote Australia: A review of the literature. Asia Pac. J. Hum. Res..

[22] Cameron P.J., Este D.C., Worthington C.A. (2012). Professional, personal and community: 3 domains of physician retention in rural communities. Can. J. Rural Med..

[23] Humphreys J., Wakerman J., Wells R., Kuipers P., Jones J., Entwistle P., Harvey P. (2007). Improving Primary Health Care Workforce Retention in Small Rural and Remote Communities: How Important is Ongoing Education and Training?.

[24] Veitch C., Battye K. (2008). Rural Health Workforce: Planning and development for recruitment and retention. Aust. Rural Health Educ. Netw..

[25] Cosgrave C. (2015). The Reality Gap—A Grounded Theory Study Investigating Turnover Intention Among Australia’s Rural-Based, Early Career Community Mental Health Professionals. Ph.D. Thesis.

[26] Vandenberg R.J., Nelson J.B. (1999). Disaggregating the motives underlying turnover intentions: When do intentions predict turnover behavior?. Hum. Relat..

[27] Xerri M. (2013). Workplace relationships and the innovative behaviour of nursing employees: A social exchange perspective. Asia Pac. J. Hum. Res..

[28] Gillham S., Ristevski E. (2007). Where do I go from here: We’ve got enough seniors?. Aust. J. Rural Health.

[29] Stagnitti K., Schoo A., Reed C., Dunbar J. (2005). Short report: Retention of allied health professionals in south-west of Victoria. Aust. J. Rural Health.

[30] Werbel J.D., Gilliland S.W., Ferris G.R. (1999). Person–environment fit in the selection process. Research in Personnel and Human Resources Management.

[31] Perkins D., Larsen K., Lyle D., Burns P. (2007). Securing and retaining a mental health workforce in Far Western New South Wales. Aust. J. Rural Health.

[32] Ziguras S., Henley K., Conron W., Catford N. (1999). Social work in mental health services: A survey of the field. Aust. Soc. Work.

[33] Crowther J., Ragusa T. (2011). Realities of mental health nursing practice in rural Australia. Issues Ment. Health Nurs..

[34] Cosgrave C., Maple M., Hussain R. (2018). Work challenges negatively affecting the job satisfaction of early career community mental health professionals working in rural Australia: Findings from a qualitative study. J. Ment. Health Train. Educ. Pract..

[35] Mills A., Millsteed J. (2002). Retention: An unresolved workforce issue affecting rural occupational therapy services. Aust. Occup. Ther. J..

[36] Ashby S.E., Ryan S., Gray M., James C. (2013). Factors that influence the professional resilience of occupational therapists in mental health practice. Aust. Occup. Ther. J..

[37] Lloyd C., King R., Bassett H. (2002). A survey of Australian mental health occupational therapists. Br. J. Occup. Ther..

[38] Strasser R., Wakegijig J., Heaney D., Abelsen B., Brandstorp H., Sigurdsson S., Berggren P. (2019). A Framework for Remote Rural Workforce Stability: Making it Work Recruit and Retain 2, Northern Periphery and Arctic Programme.

[39] O’Toole K., Schoo A., Hernan A. (2010). Why did they leave and what can they tell us? Allied health professionals leaving rural settings. Aust. Health Rev..

[40] Strasser R., Kam S.M., Regalado S.M. (2016). Rural health care access and policy in developing countries. Annu. Rev. Public Health.

[41] Russell D., Zhao Y., Guthridge S., Ramjan M., Jones M.P., Humphreys J., Wakerman J. (2017). Patterns of resident health workforce turnover and retention in remote communities of the Northern Territory of Australia, 2013–2015. Hum. Res. Health.

[42] Urquhart H. The Role of Community Engagement in Improving Recruitment and Retention of Health Care Workers in Rural and Remote Areas. https://www.uhi.ac.uk/en/t4-media/one-web/university/research/srhp/making-it-work/Miw-Community-Engagment-Paper-(final)-V2-Oct-2018.pdf.

[43] Veitch C., Harte J., Hays R., Pashen D., Clark S. (1999). Community participation in the recruitment and retention of rural doctors: Methodological and logistical considerations. Aust. J. Rural Health.

[44] Martiniuk A., Colbran R., Ramsden R., Karlson D., O’Callaghan E., Lowe E., Edwards M., Bagnulo S., Rothnie I., Hardaker L. (2019). Hypothesis: Improving literacy about health workforce will improve rural health workforce recruitment, retention and capability. Hum. Res. Health.

[45] Mellor D., Stokes M., Firth L., Hayashi Y., Cummins R. (2008). Need for belonging, relationship satisfaction, loneliness, and life satisfaction. Personal. Individ. Differ..

[46] Hummon D.M. (1992). Community attachment. Place Attachment.

[47] Hancock C., Steinbach A., Nesbitt T., Adler S., Auerswald C. (2009). Why doctors choose small towns: A developmental model of rural physician recruitment and retention. Soc. Sci. Med..

[48] Manahan C.M., Hardy C.L., MacLeod M.L.P. (2009). Personal characteristics and experiences of long-term allied health professionals in rural and northern British Columbia. Rural Remote Health.

[49] Pierce J. (2017). How We Came to Stay: Narratives of Social Workers in Remote Northern Regions of British Columbia. Ph.D. Thesis.

[50] Kulig J., Stewart N., Penz K., Forbes D., Morgan D., Emerson P. (2009). Work setting, community attachment, and satisfaction among rural and remote nurses. Pub. Health Nurs..

[51] Godwin D.M., Hoang H., Crocombe L.A., Bell E. (2014). Dental practitioner rural work movements: A systematic review. Rural Remote Health.

[52] Erikson E. (1982). The Life Cycle Completed: A Review.

[53] Levinson D. (1978). The Seasons of a Man’s Life.

[54] Valliant G. (1993). The Wisdom of the Ego: Sources of Resilience in Adult Life.

[55] Auer K., Carson D. (2010). How can general practitioners establish ‘place attachment’ in Australia’s Northern Territory? Adjustment trumps adaptation. Rural Remote Health.

[56] Cosgrave C., Malatzky C., Gillespie J. (2019). Social determinants of rural health workforce retention: A scoping review. Int. J. Environ. Res. Public Health.

[57] Myroniuk L., Adamiak P., Bajaj S., Myhre D. (2016). Recruitment and retention of physicians in rural Alberta: The spousal perspective. Rural Remote Health.

[58] Albion M.J., Fogarty G.J., Machin M.A., Patrick J. (2008). Predicting absenteeism and turnover intentions in the health professions. Aust. Health Rev..

[59] Gallego G., Chedid R.J., Dew A., Lincoln M., Bundy A., Veitch C., Bulkeley K., Brentnall J. (2015). Who are they and what do they do? Profile of allied health professionals working with people with disabilities in rural and remote New South Wales. Aust. J. Rural Health.

[60] Woodend A.K., Cooper J., Buske L., Marcus L., Chauhan T.S., Little L., Teperman L., Adams O. (2005). Retaining pharmacists in rural Canada. Canad. Pharm. J..

[61] Kulig J., Townshend I., Kosteniuk J., Karunanayake C., Labrecque M.E., MacLeod M.L. (2018). Perceptions of sense of community and community engagement among rural nurses: Results of a national survey. Int. J. Nurs. Stud..

[62] Allan J. (2011). Mining’s relocation culture. Int. J. Sociol. Soc. Policy.

[63] Reagan E.M., Hambacher E., Schram T., McCurdy K., Lord D., Higginbotham T., Fornauf B. (2019). Place matters: Review of the literature on rural teacher education. Teach. Teach. Educ. Int. J. Res. Stud..

[64] Humphreys J., Wakerman J., Wells R., Kuipers P., Jones J.A., Entwistle P. (2008). “Beyond workforce”: A systemic solution for health service provision in small rural and remote communities. Med. J. Aust..

[65] Allan J., Ball P. (2008). Developing a competitive advantage: Considerations from Australia for the recruitment and retention of rural and remote primary health workers. Aust. J. Prim. Health.

[66] Beer M., Spector B.A., Lawrence P.R., Mills D.Q., Walton R.E. (1984). Managing Human Assets.

[67] Perkins D., Farmer J., Salvador-Carulla L., Dalton H., Luscombe G. (2019). The Orange Declaration on rural and remote mental health. Aust. J. Rural Health.

